# Postoperative chemoradiotherapy is superior to postoperative chemotherapy alone in squamous cell lung cancer patients with limited N2 lymph node metastasis

**DOI:** 10.1186/s12885-019-6141-z

**Published:** 2019-10-30

**Authors:** Liyu Su, Mingqiu Chen, Huiyan Su, Yaqing Dai, Shaoxing Chen, Jiancheng Li

**Affiliations:** 1 Department of Radiation Oncology, Fujian Cancer Hospital & Fujian Medical University Cancer Hospital, Fuzhou, 350014 Fujian China; 2 Fujian Medical University, Fujian, 350122 China; 3 Department of Oncology, Fujian Cancer Hospital & Fujian Medical University Cancer Hospital, Fuzhou, 350014 Fujian China; 4 Fujian Provincial Platform for Medical Laboratory Research of First Affiliated Hospital, Fujian, China; 5 Department of Radiation Oncology, Fujian Children’s Hospital, Fujian, China; 6 Department of Radiation Oncology, The First Affiliated Hospital of Xiamen University, Fujian, China; 7 Department of Radiation Oncology, The 175th Hospital of PLA (The Chinese People’s Liberation Army), Fujian, China

**Keywords:** N2 lymph node metastasis, Non-small-cell lung cancer, Postoperative chemoradiotherapy, Survival

## Abstract

**Background:**

The aim of the present study was to assess the efficacy of postoperative chemoradiotherapy (POCRT) following surgery in non-small-cell lung cancer patients with N2 lymph node metastasis (N2-NSCLC).

**Methods:**

The clinical data of patients with N2-NSCLC treated with POCRT or postoperative chemotherapy (pCT) alone were retrospectively collected and reviewed. The overall survival (OS) rates were analyzed utilizing the Kaplan-Meier method and compared by the log-rank test. Cox regression analysis was used to determine factors significantly associated with survival. Propensity score matching (PSM) analysis was used to compensate for differences in baseline characteristics and OS was compared after matching.

**Results:**

Between 2004 and 2014, a total of 175 patients fulfilled the inclusion criteria, 60 of whom were treated with POCRT, while 115 were administered pCT. The 1, 3 and 5-year OS rates in the POCRT and pCT groups were 98.3 vs. 86.1%, 71.7 vs. 53.0% and 45.7 vs. 39.0%, respectively (*P* = 0.019). Compared with pCT, POCRT improved OS in patients with squamous cell subtype (*P* = 0.010), no lymphovascular invasion (*P* = 0.006), pN2a (P = 0.006) or total number of metastatic lymph nodes ≤7 (*P* = 0.016). After PSM, these survival differences between POCRT and pCT remained significant in patients with squamous cell lung cancer (*P* = 0.010).

**Conclusions:**

POCRT following complete resection may be beneficial for patients with squamous cell lung cancer, particularly those with limited nodal involvement.

## Background

Lung cancer remains the most common type of cancer and the leading cause of cancer-related mortality worldwide, with 1.8 million deaths predicted and 2.1 million new lung cancer cases in 2018 [1] and an increasing estimated cancer incidence, expected to reach three million by 2035 [2]. World Health Organization (WHO) divides lung cancer into small-cell lung cancer (SCLC) and non-small-cell lung cancer (NSCLC) [3]. NSCLC accounts for > 80% of all lung cancer cases and it includes two major pathological subtypes: Squamous cell (epidermoid) carcinoma and non-squamous cell carcinoma (including adenocarcinoma, large-cell carcinoma and other subtypes) [4].

Surgical resection is currently the mainstay of definitive treatment for localized NSCLC. In addition, postoperative chemotherapy (pCT) is considered as the standard postoperative treatment for NSCLC patients with metastases to the lymph nodes [5]. However, even when administered with bimodal treatment (BMT, surgery and pCT) strategies, the prognosis of patients with metastatic lymph nodes (MLN) remains dismal (< 25% at 5 years) [6], which is mainly due to a high (up to 30%) local tumor failure rate as the first site of recurrence [7]. Thus, it has come to be considered that postoperative radiotherapy (PORT) should be added to BMT to improve local control and survival, although this may not be the case, as several clinical trials confirmed that POCRT did not improve the survival of patients with N1 stage disease after complete (R0) resection [8]. Particularly for patients with metastases to the ipsilateral mediastinal lymph nodes (N2-NSCLC), the role of PORT following BMT remains controversial due to its variable response rates and effectiveness [9].

In the present study, the clinical data of patients with N2-NSCLC treated with POCRT or pCT following surgery were retrospectively collected and analyzed to explore the status of POCRT in N2-NSCLC.

## Methods

### Patient selection criteria

This retrospective study was approved by Fujian Province Cancer Hospital Institutional Review Board (No. KT-2018-015-01). All patients provided written informed consent prior to treatment, and all information was anonymized prior to analysis.

The eligibility and exclusion criteria for the present retrospective study were as follows: Primary histologically proven NSCLC, good performance status (Eastern Cooperative Oncology Group performance status score (ECOG PS) ≤2), complete pretreatment workup and follow-up data, and without other concomitant medical conditions that required treatment, initially treated with curative surgery followed by chemotherapy or radiotherapy, pathological stage TanyN2M0 (pTanyN2M0). Patients who survived < 1 month after surgery were considered as surgical fatalities and were excluded from the present study.

The pTNM stage was re-determined according to the 8th American Joint Committee on Cancer TNM staging system [10] based on the data of the surgical pathology specimen. N2 was subclassified into N2 at a single station without N1 involvement (‘skip’ metastasis, N2a1), N2 at a single station with N1 involvement (N2a2), and N2 at multiple stations (N2b) [11].

### Treatment

All enrolled patients were initially treated with thoracotomy, including wedge or sleeve resection, lobectomy and pneumonectomy, by minimally invasive or conventional surgery. The initiation of pCT started no later than 2 weeks after the operation. The regimens of pCT in the present study included a two-drug combination chemotherapy regimen based on cisplatin and were administered for at least 2 cycles of full-dose chemotherapy. Chemotherapy regimen includes NP (vinorelbine 25 mg/m^2^ dl, d8 + cisplatin 25 mg/m^2^ dl-3), GP (gemcitabine 1250 mg/m^2^ dl, d8 + cisplatin 25 mg/m^2^ dl-3), TP (paclitaxel 135 mg/m^2^ d1+ Cisplatin 25 mg/m^2^ dl-3), DP (docetaxel 75 mg/m^2^ dl + cisplatin 25 mg/m^2^ dl-3), PC (pemetrexed 500 mg/m^2^ dl + cisplatin 25 mg/m^2^ dl-3,only for non-squamous cell carcinoma), 21-28d/cycle. Carboplatin was alternatively used in case of intolerance to cisplatin. Adjustments to the pCT time intervals and dose intensities were similar to our previous study [12].

POCRT was executed sequentially or sandwiched with pCT with three-dimensional conformal or intensity-modulated radiotherapy technique. The targets, including clinical target volume (CTV), planned target volume (PTV) and organs at risk (OARs) of radiotherapy. The CTV should include the bronchial stump and the high-risk lymphatic drainage area. The PTV was defined as the CTV plus a 0.5 or 0.6 cm margin for setup uncertainty and respiratory motion. The prescribed dose is defined as the dose received by 95% PTV. Dose limitation for organ-at-risk was defined: lungs V20 ≤ 25%, lungs V5 < 60%, unilateral lung V20 ≤ 45%. Heart V30 < 40%, V40 < 30%, mean dose ≤30Gy, esophagus V50 < 50%, and < 45 Gy for maximum spinal cord dose. The target dose and the dose limitations of OARs were defined and adjusted as described in our previous study [12]. The median dose to CTV was 5000 (range 4400-6000) cGy, with 180–200 cGy per fraction.

### Surveillance and statistical analysis

The follow-up schedule for patients was as previously reported [12]. In brief, patients were evaluated every 3 months for the first 2 years after surgery, every 6 months for the next 3 years, and once annually thereafter. All patient outcomes were evaluated in April 2018. The primary endpoint was OS. The OS was calculated from the date of diagnosis to the date of death, or the date of the last follow-up.

Data were analyzed using SPSS version 24.0 (IBM Corp., Armonk, NY, USA). Survival curves were produced using the Kaplan-Meier estimator method and compared with the log-rank test. Univariable and multivariable analyses of clinical characteristics, including sex, age, tumor location, pT stage, pN2 subclass, total number of MLNs, histopathological type, lymphovascular invasion, radiotherapy dose of CTV, regimens and cycles of pCT and surgical modality, associated with OS, were performed using the Cox proportional hazards model. Confidence intervals (CIs) represented 95% lower and upper limits.

Propensity score matching (PSM) analyses were used to compensate for differences in baseline characteristics between the POCRT and pCT groups to confirm the survival difference. First, all available patient and tumor variables were compared using the χ^2^ test. Next, a propensity score was calculated using a logistic regression with the imbalanced variables that were statistically significantly correlated with OS on multivariate analysis. Finally, all analyses regarding OS were adjusted based on the generated propensity score [13]. Pearson’s χ^2^ test was subsequently performed to compare the differences between the POCRT and pCT groups after matching.

## Results

### Patient characteristics

Between September 2004 and December 2014, a total of 3262 surgically treated patients with NSCLC were reviewed. A total of 175 patients fulfilled the inclusion criteria, of whom 115 patients were administered pCT and 60 patients received POCRT. No significant differences in clinical characteristics were identified between the two groups, with the exception of surgical modality and total number of chemotherapy cycles (Table 1), which did not affect patient survival in the subsequent multivariate analyses.

**Table 1:** Clinical characteristics of patients before and after matching

Characteristics	Pre-matched			Matched		
	POCRT	pCT	p	POCRT	pCT	p
Gender			0.523			0.385
Male	42	75		42	33	
Female	18	40		18	20	
Median age (y, range)	58 (37–74)	56 (33–75)	0.222	58 (37–74)	57 (41–73)	0.217
ECOG scoring			0.572			0.149
0	18	36		18	21	
1	42	77		42	30	
2	0	2		0	2	
Position			0.636			0.190
Central	30	62		30	20	
Periphery	30	53		30	33	
Vessel invasion						
Positive	16	28	0.737	16	15	0.846
Negative	44	87		44	38	
pT stage			0.099			0.067
1	13	16		13	16	
2	35	58		35	35	
3	8	19		8	1	
4	4	22		4	1	
Operation modality			0.044			0.674
Wedge or Sleeve	4	2		4	2	
Lobectomy	54	99		54	48	
Pneumonectomy	2	14		2	3	
Pathology			0.582			0.261
SCC	18	30		18	11	
Non-SCC	42	85		42	42	
pN stage			0.924			0.646
N2a1	13	26		13	9	
N2a2	18	37		18	20	
N2b	29	52		29	24	
Chemotherapy cycles (range)	4 (2–6)	3 (2–6)	0.0001	4 (2–6)	4 (2–6)	0.143
Total number of MLNs (range)	4 (1–28)	4 (1–26)	0.536	4 (1–28)	4 (1–23)	0.333
CTV dose (range)	5000 (4400–6000)	–		5000 (4400–6000)	–	

### Survival analysis in the entire cohort

At the last follow-up, 57 patients remained alive and 118 patients had died, of whom 87 patients had succumbed to the disease (10 to locoregional recurrence, 34 to distant metastasis and 43 to both) and 31 patients had died from unknown causes (Table 2).

**Table 2:** Failure Pattern and survival

	POCRT	pCT	Total	p
Pattern of failure, n (%)				0.047
Locoregional alone	2	8	10	
Locoregional and distant	13	30	43	
Distant alone	16	18	34	
unknown	5	26	31	
OS rates (%)				0.019
1- year	98.3	86.1	90.3	
3- year	71.7	53.0	60.0	
5- year	45.7	39.0	41.2	

**Fig. 1:**
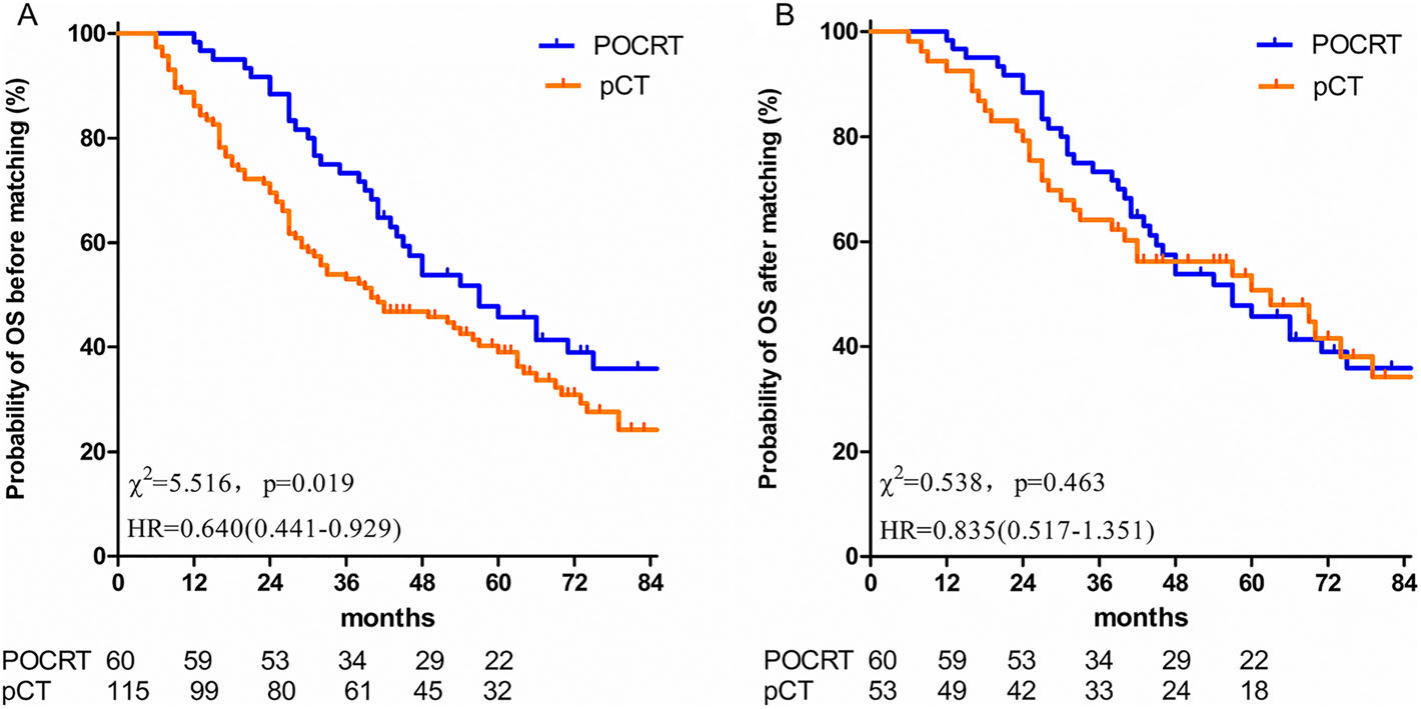
Comparison of OS between pCT alone and POCRT before and after matching. **a**. Comparison of OS between pCT alone and POCRT before matching. **b**. Comparison of OS between pCT alone and POCRT after matching. OS, overall survival; pCT, postoperative chemotherapy; POCRT, postoperative chemoradiotherapy

The median follow-up time in the entire cohort and in the surviving patients was 48 (6–128) and 68 (38–128) months, respectively. The 1, 3 and 5-year OS rates for the entire cohort were 90.3, 60.0 and 41.2%, respectively. The 1, 3 and 5-year OS rates in the POCRT and pCT groups were 98.3 vs. 86.1%, 71.7 vs.53.0% and 45.7 vs. 39.0%, respectively (*P* = 0.019) (Fig. 1a, Table 2).

**Fig. 2:**
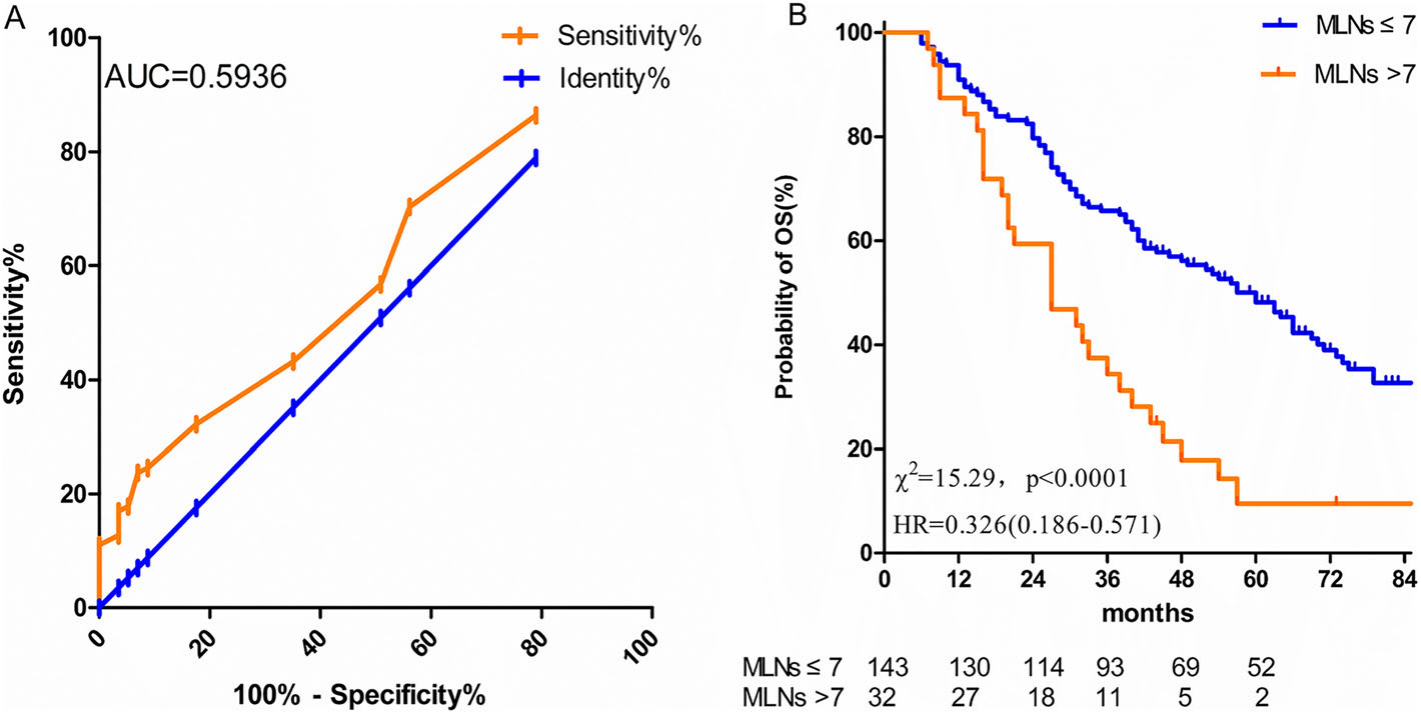
**a**.The ROC curve of the cut-off number of MLNs. **b**. Comparison of OS between ≤7 MLNs and > 7 MLNs

Univariate and multivariate analyses indicated that T stage, total number of MLNs and POCRT were independent factors affecting OS (Table 3). The ROC curve of the total number of MLNs was applied to identify the cut-off number that was most significantly correlated with patient survival using the area under the curve (AUC) (Fig. 2a), indicating that patients with > 7 MLNs had a significantly inferior survival compared with patients with ≤7 MLNs (*P* = 0.0001) (Fig. 2b).

**Table 3:** Prognostic factors by univariate and multivariate analysis

Prognostic factors	Univariate		Multivariable	
	p	HR (95.0% CI)	p	HR (95.0% CI)
Gender	0.135	1.014 (0.991–1.037)		
Age	0.245	0.736 (0.492–1.100)		
ECOG	0.214	1.269 (0.872–1.848)		
Tumor location	0.110	0.742 (0.514–1.070)		
Operation modality	0.006	2.040 (1.230–3.382)		
Pathology type	0.912	1.023 (0.686–1.525)		
pT stage	0.011	1.287 (1.059–1.565)	0.024	1.264 (1.031–1.550)
pN stage	0.929	1.011 (0.798–1.280)		
Total number of MLNs	0.0001	1.085 (1.047–1.124)	0.0001	1.090 (1.053–1.130)
Vessel invasion	0.821	0.951 (0.617–1.467)		
Total Chemotherapy cycles	0.019	0.797 (0.659–0.964)		
POCRT	0.021	0.628 (0.423–0.932)	0.012	0.601 (0.403–0.895)

### Survival analysis between POCRT and pCT in various subgroups

**Fig. 3:**
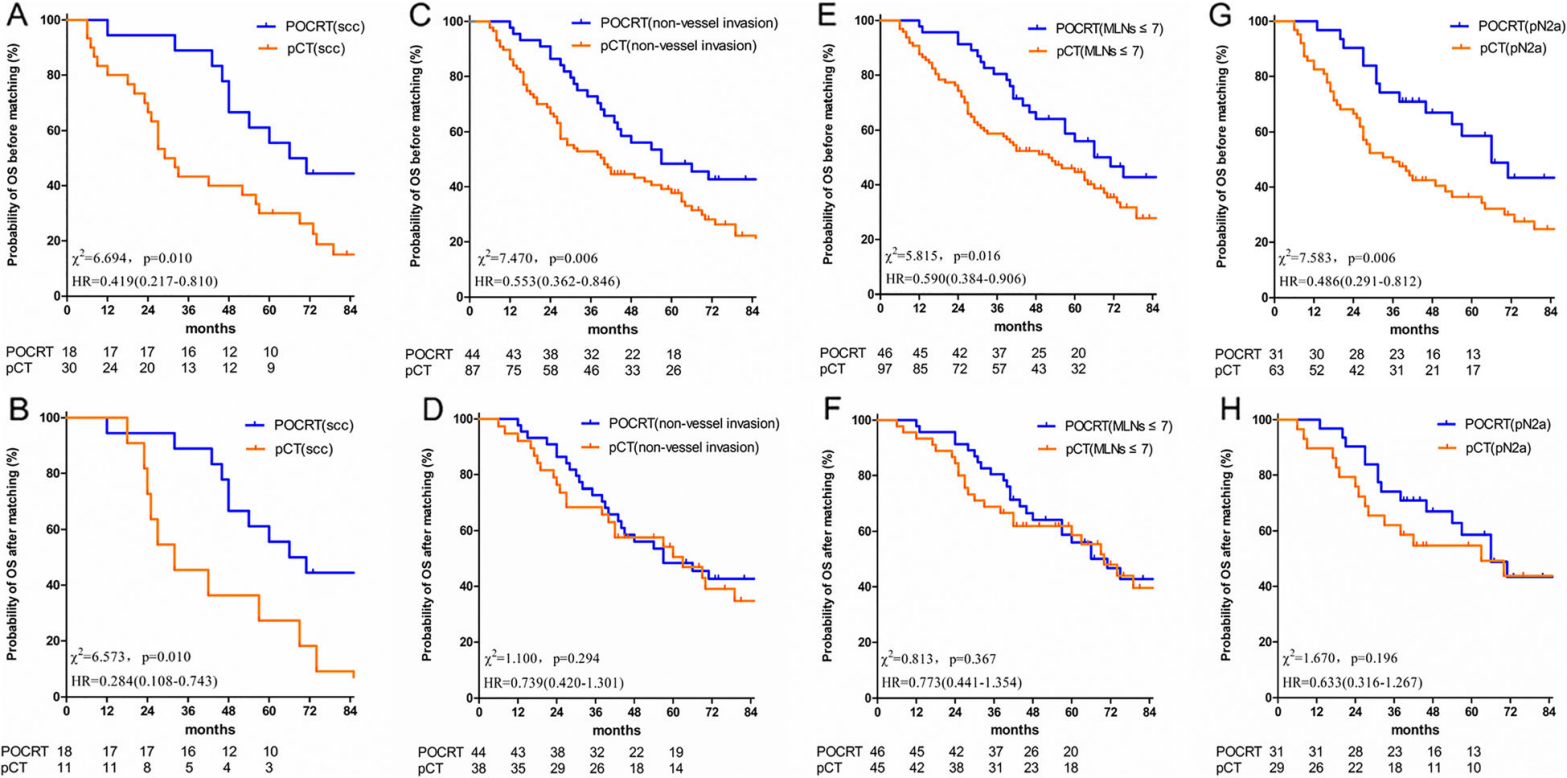
OS in various subgroups of independent significant factors. OS, overall survival. **a**. Comparison of OS between pCT alone and POCRT in patients with squamous cell carcinoma before matching. **b**. Comparison of OS between pCT alone and POCRT in patients with squamous cell carcinoma after matching. **c**. Comparison of OS between pCT alone and POCRT in patients with without lymphovascular invasion before matching. **d**. Comparison of OS between pCT alone and POCRT in patients with without lymphovascular invasion after matching. **e**. Comparison of OS between pCT alone and POCRT in patients with ≤7 MLNs before matching. **f**. Comparison of OS between pCT alone and POCRT in patients with ≤7 MLNs after matching. **g**. Comparison of OS between pCT alone and POCRT in patients with pN2a before matching. **h**. Comparison of OS between pCT alone and POCRT in patients with pN2a after matching. OS, overall survival; pCT, postoperative chemotherapy; POCRT, postoperative chemoradiotherapy; scc, squamous cell carcinoma; non-vessel invasion, without lymphovascular invasion; MLNs≤7, metastatic lymph nodes≤7; pN2a, Combine pN2a1 and pN2a2, N2 at a single station without N1 involvement (‘skip’ metastasis, N2a1), N2 at a single station with N1 involvement (N2a2)

To identify patients who may benefit from POCRT, exploratory subgroup analyses were conducted among various patient subgroups, particularly of the abovementioned significant independent prognostic factors. The results demonstrated that, compared with the corresponding subgroups, POCRT benefited patients with squamous cell histology, without lymphovascular invasion, ≤7 MLNs or N2a (Fig. 3a, c, e and g). Although the survival of patients with T4 stage also differed between POCRT and pCT, the conclusion was not robust, as the number of patients with T4 in the present study was limited.

To balance the bias due to the retrospective nature of this study, PSM based on the clinical baseline characteristics, including age, gender, surgical modality, pathology, lymphovascular invasion, pT stage, pN stage, total number of MLNs, regimens and cycles of chemotherapy, was conducted. Following PSM, a total of 113 events were identified in both the POCRT and pCT groups, with 60 and 53 patients in each group, respectively. Following PSM, the 1, 3 and 5-year OS rates in the POCRT and pCT groups were 98.3 vs. 88.7%, 71.7 vs.62.3% and 45.7 vs. 50.7%, respectively (*P* = 0.463) (Fig. 1b). And the survival differences between POCRT and pCT in the various subgroups were not statistically significant, except in patients with squamous cell lung cancer (Fig. 3b, d, f and h).

## Discussion

Even with the development of molecular targeted treatment for NSCLC, pCT remains the standard postoperative adjuvant treatment for NSCLC patients with MLNs [5], whereas the additive effects of delivering radiotherapy (RT) to MLN-NSCLC patients treated with BMT have not been established [9]. Burdett et al. conducted a meta-analysis of randomized trials and demonstrated that, compared with pCT alone, POCRT failed to confer a survival benefit in NSCLC patients with either N0, N1 or N2 disease [14]. However, the greatest limitation of Burdett’s report was that patients enrolled in that study were treated with outdated radiation equipment and techniques, which ultimately contributed to a negative outcome as a result of RT-related cardiac and pulmonary toxicity [5]. Multiple subsequent studies have been conducted to explore the role of POCRT with contemporary RT techniques in resectable NSCLC. Unfortunately, the results demonstrated that even POCRT using modern technology did not confer a survival benefit to patients with NSCLC, but instead increased the relative risk of death [8].

However, contrary to abovementioned studies, Lally et al. conducted a landmark meta-analysis using the Surveillance, Epidemiology and End Results Database and reported that, despite the fact that survival was not increased in patients with N0 and N1 disease, N2-NSCLC patients achieved a notable OS improvement from POCRT with modern technology [15]. Then, several comparable studies confirmed the advantage of POCRT in N2-NSCLC [16]. Similarly, in the present retrospective study, compared with pCT, POCRT achieved a significant survival benefit in N2-NSCLC patients. To make our conclusion more robust, PSM was conducted to compensate the selection bias of the present retrospective study. Following PSM, POCRT still clearly demonstrated superior survival compared with pCT, indicating that POCRT should be considered for patients with N2-NSCLC, despite the fact that no randomized clinical trials have been conducted to validate it thus far [17].

Due to the heterogeneous nature of N2-NSCLC, some studies proposed that POCRT should only be considered for a certain N2 subgroup rather than for all N2-NSCLC patients [9]; however, the criteria for classifying N2 disease into subcategories had not been established. Asamura et al. proposed staging N2 into pN2a1, pN2a2 and pN2b subgroups based on the combination of MLN location, number and absence versus presence of skip metastases [11], which had been reported to estimate a more accurate prognosis in N2-NSCLC. To identify which N2 subgroup would benefit from POCRT, a stratified analysis based on Asamura’s N2 staging system performed in the present study indicated that, compared with pCT, patients with N2a (whether N2a1 or N2a2) treated with POCRT achieved a significantly better survival.

Considering that the total number of MLNs represented a strong independent prognostic factor in NSCLC [18], a stratified analysis according to the total number of MLNs was conducted to determine which patient subgroup would benefit from POCRT in the present study. The results demonstrated that patients with ≤7 MLNs, contrary to those with > 7 MLNs, gained a significant survival benefit from POCRT compared with pCT.

It is well-known that lymphovascular invasion, which forebodes a high risk of nodal metastasis, is an independent factor affecting survival in early-stage NSCLC patients [19–21]. However, contrary to its prominent role in early-stage lung cancer, lymphovascular invasion was not found to be significantly correlated with survival in the present study. This finding indicated that, in advanced N2-NSCLC, the effect of lymphovascular invasion on survival may be offset by the effect of N2. In addition, the stratified analysis demonstrated that, compared with pCT, patients without lymphovascular invasion gained a more prominent survival benefit from POCRT.

Taken together, the abovementioned results lead to the conclusion that POCRT may improve the survival of N2-NSCLC patients with limited nodal involvement. An explanation of the results may be that patients with more extensive nodal spread had a high frequency of distant metastases [22] and the local control achieved with POCRT cannot be translated into long-term survival.

Although surgery is indicated in selected T4 N0–1 patients, the efficacy of surgery in patients with T4 N2 disease has not been convincing [23]. Patel et al. recommended that only T4 N2 patients with good performance status and minimal N2 nodal involvement (single-station, microscopic) should be considered for surgery [24]. However, as patients with T4 and minimal N2 are rare, the benefit of POCRT for T4 N2 patients had not been previously reported. The present study demonstrated that patients with T4 stage had a significantly inferior survival compared with stage T1, T2 and T3. However, the conclusion was not convinced owing to the sample of patients with T4 disease was too small in the current study.

Previous studies demonstrated that central tumor location is associated with a higher rate of surgical resection margin positivity compared with peripheral tumor location, and such patients may benefit from POCRT [25]. However, to the best of our knowledge, there is no study on POCRT for patients with centrally located tumor undergoing R0 resection. The present study indicated that survival did not differ significantly between central and peripheral tumor location, whether in the entire cohort or in the PSM matched cases. Therefore, tumor location was not found to be a risk factor for N2-NSCLC and should not considered an indicator for POCRT.

The management of lung adenocarcinoma had dramatically changed over the past decade with the introduction of targeted therapeutic agents for genotypical selection [26, 27]. By contrast, progress in squamous cell lung cancer treatment has been modest, and there has yet to be a successful application of targeted therapy in this disease [28]; therefore, patients with squamous cell lung cancer have been receiving the same conventional treatment for the last decade. In the present study, patients with squamous cell lung cancer achieved a notable survival benefit from POCRT, whether in the entire cohort or in the matched cases.

Therefore, POCRT may be specifically recommended to N2 patients with squamous cell lung cancer due to the current lack of effective targeted therapies. However, as the present study is the first to demonstrate that POCRT confers a survival benefit in squamous cell lung cancer based on a limited patient sample, this recommendation should be interpreted with caution as it requires validation by further clinical trials.

## Conclusions

POCRT following complete resection may be beneficial for patients with squamous cell lung cancer, particularly those with limited N involvement. Due to the limitations of the present study, including the retrospective design with inherent biases, the small sample enrolled, the lack of unified chemotherapy regimens and the assessment of OS alone, the results of our investigation must be interpreted with caution.

## Supplementary information

**Additional file 1.** Associated Data.The file included the data of the treatment of 175 NSCLC patients. The treatment group was divided into 2 subgroups(0 for pCT and 1 for POCRT).The gender, age, ECOG scoring ,tumor position, Vessel invasion, T stage, Operation modality, Pathology, pN stage, Chemotherapy cycles, Total number of MLNs and CTV dose were divided into different subgroups according to the details list in the Table 1. The OS was calculated from the date of diagnosis to the date of death, or the date of the last follow-up. The survival group was divided into 2 subgroups (0 for survival and 1 for death). (XLSX 35 kb)

## Data Availability

The data used to support the findings of this study are included with the article and Additional file 1.

## Abbreviations

AUC: Area under the curve
BMT: Bimodal treatment
CIs: Confidence intervals
CTV: Clinical target volume
ECOG PS: Eastern Cooperative Oncology Group performance status score
MLNs: Metastatic lymph nodes
N2-NSCLC: Non-small-cell lung cancer patients with N2 lymph node metastasis
NSCLC: Non-small-cell lung cancer
OARs: Organs at risk
OS: Overall survival
pCT: Postoperative chemotherapy
POCRT: Postoperative chemoradiotherapy
PORT: Postoperative radiotherapy
PSM: Propensity score matching
PTV: Planned target volume
RT: Radiotherapy
SCLC: Small-cell lung cancer
WHO: World Health Organization
